# Sublingual microcirculatory alterations in Chagas disease: an observational study in an endemic rural population

**DOI:** 10.1590/0074-02760240018

**Published:** 2024-08-02

**Authors:** Jorge Emilio De All, Juan Francisco Caminos Eguillor, Simón Marcelo Cohen, Héctor Freilij, Arnaldo Dubin

**Affiliations:** 1 Asociación Cuerpo & Alma, Ciudad Autónoma de Buenos Aires, Argentina; 2 Sanatorio Otamendi, Ciudad Autónoma de Buenos Aires, Argentina; 3 Universidad Nacional de La Plata, Facultad de Ciencias Médicas, Cátedras de Farmacología Aplicada y Terapia Intensiva, Provincia de Buenos Aires, Argentina; 4 Hospital de Niños Ricardo Gutiérrez, Servicio de Parasitología y Enfermedad de Chagas, Ciudad Autónoma de Buenos Aires, Argentina

**Keywords:** Chagas disease, microcirculation, angiogenesis, microvascular density

## Abstract

**BACKGROUND:**

Chagas disease is a systemic illness with widespread microvascular involvement. Experimental and clinical studies suggest that functional and structural microcirculatory abnormalities might be relevant to the disease progression.

**OBJECTIVES:**

To show the presence of sublingual microcirculatory alterations in patients with chronic Chagas disease.

**METHODS:**

This was a cross-sectional study including adult patients with serologic diagnosis of Chagas disease (n = 41) and control volunteers with negative serology (n = 38), from an endemic rural population. Study participants underwent clinical, electrocardiographic, echocardiographic, and sublingual videomicroscopic assessment. Videos were acquired by a sidestream-dark-field (SDF) imaging device and evaluated by a software-assisted analysis (AVA 3.2 software).

**FINDINGS:**

Most of Chagas disease patients were in the indeterminate phase (n = 34) and had lower heart rate and more echocardiographic abnormalities than control group (50 vs. 26%, p = 0.03). They also exhibited higher small microvessels total and perfused vascular density (20.12 ± 2.33 vs. 19.05 ± 2.25 and 20.03 ± 2.28 vs. 19.01 ± 2.25 mm/mm^2^, p < 0.05 for both). Other microvascular variables did not differ between groups.

**MAIN CONCLUSIONS:**

Patients with chronic Chagas disease exhibited increases in sublingual total and perfused microvascular density. Angiogenesis might be the underlying mechanism. The videomicroscopic assessment of mucosal sublingual microcirculation might be an additional tool in the monitoring of Chagas disease.

Chagas disease (CD) is a potentially life-threatening illness caused by the protozoan parasite *Trypanosoma cruzi*. While an estimated 6 to 7 million people are infected with *T. cruzi* in Latin American countries, 75 million could be at risk of infection worldwide.^1^ Following an acute phase characterised by high parasitaemia and intense inflammatory response, a chronic phase begins after 1-2 months. Most patients remain asymptomatic throughout life ─ the so-called indeterminate form of the disease. However, about 30% of them suffer from cardiac disorders, and up to 10% exhibit digestive, neurological, or mixed manifestations.^2^

The chronic disease is linked to parasite persistence at tissue level. For example, in chronically infected rodents, *T. cruzi* DNA is detectable in almost all organs.^3^ Similarly, patients with chronic CD exhibit a widespread distribution of amastigotes in extracardiac tissues.^4^ Clinical manifestations likely result from complex interactions between the parasite and the immunological and the inflammatory response, with microcirculation emerging as a key target.^5^

The microvascular involvement might play a pivotal role in the progression of the disease. Evidence from pathological, experimental, and clinical studies suggests that functional and structural microvascular abnormalities might lead to myocardial ischemia and contribute to the pathogenesis of Chagas heart disease.^6^ While the heart is the main organ affected, many microcirculatory vascular beds can be compromised during the different phases of the disease. In animal models of acute disease, decreases in cerebral functional capillary density^7^ and in cremaster red blood cell velocity^8^ were described. Conversely, angiogenic phenomena and increases in vascular density were found in cardiac^9^ and extracardiac tissues.^10^ Furthermore, patients with chronic CD exhibited increased lingual vascular density.^11^

Over the past two decades, a large body of clinical evidence has emerged regarding the relevance of microcirculation in the pathophysiology of critical illness, especially in sepsis. Most advances are based on the assessment of sublingual microcirculation using hand-held videomicroscopy.^12^

Given the systemic characteristics of CD and its extensive microvascular abnormalities, we hypothesised that the sublingual mucosa could exhibit microvascular disorders in chronic CD patients. To test this hypothesis, we studied subjects infected and non-infected with *T. cruzi*, from a rural population with high prevalence of infection, in Chaco, Argentina.^13^ An additional goal was to evaluate the relationship of alterations in sublingual microcirculation with the presence of heart disease and the parasite load.

## SUBJECTS AND METHODS

This study was approved by the Ethics in Research Committee of the Argentinean Society of Cardiology (approval date: October 2, 2017). Written informed consent was obtained from each participant.

*Design* - Cross-sectional study.

**Fig. 1:**
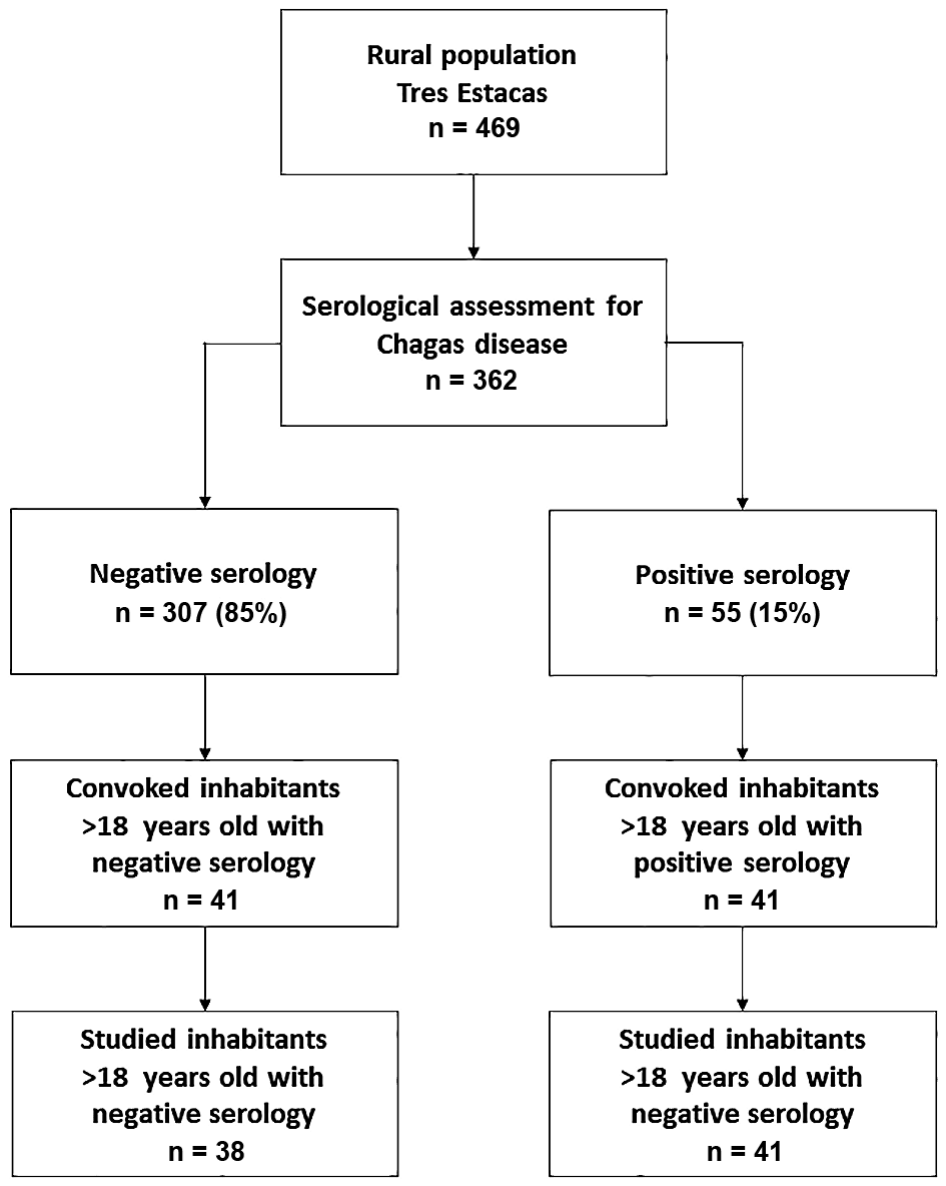
flowchart of the study.

**Fig. 2:**
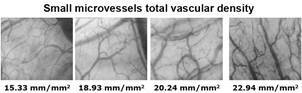
typical images corresponding to different values of small microvessels total vascular density sublingual microcirculation.

*Study setting* - The study conducted at Tres Estacas, a rural village located 50 kilometres west of the city of Charata, in the southwest of the Province of Chaco, Argentina. The area is made up of a village with a health post in charge of a nursing assistant and a school with primary and secondary level. The logistics operation for the evaluation of the patients was carried out by air transfer from the city of Buenos Aires to the city of Charata. The work team was composed of two aircraft pilots, one cardiologist, two intensivists trained in the acquisition of sublingual microcirculatory images by videomicroscopy, one internal medicine specialist and three administrative assistants. Likewise, videomicroscopic, echocardiographic and electrocardiographic equipment, along with disposable materials was transported. Daily, the team travelled the 50 kilometres from Charata to Tres Estacas in a four-wheel drive truck along a rural road in very poor condition [Supplementary data (Figs 1-4)].

Before the recruitment, a household and peri-domicile entomological survey was carried out to detect triatomes, using the man-hour method. The workers who carried out the task were trained by staff from the National Chagas Disease Program. All the houses in the community were sprayed with beta-cypermethrin.

*Subjects* - We studied adult subjects (≥ 18 years old) of both genders, with a serologic diagnosis of CD and a control group with negative serology, from a rural population.

*Measurements* - Serological tests were carried out on all residents over 18 years of age who attended the local school, through a call made through the civil association Cuerpo & Alma and local health agents. Blood samples of 5 mL were extracted by venepuncture at the level of the forearm and centrifuged at 3000 rpm for 15 min. The serum was stored at 4ºC. Indirect immunofluorescence assay (IFA) performed by fixed epimastigotes (Laboratorios Chaqueños SA, Resistencia, Chaco, Argentina) and anti-human immunoglobulin G-fluorescein conjugate (Biocientifica, Buenos Aires, Argentina), enzyme linked immunosorbent assay (ELISA) (The Chagatest ELISA test v.3.0, Wiener Lab, Rosario, Argentina), and indirect hemagglutination (IHA) (HAI Chagas; Polychaco SAIC, Buenos Aires, Argentina) were used as serological methods for the diagnosis of CD. We considered cut-off values for the IFA and the IHA ≥ 1/32. The diagnosis of CD required at least two out of three positive serological tests.

Patients with positive serology were evaluated using real time polymerase chain reaction (RT-PCR) to determine parasite load [Supplementary data (Table I)]. The assay used has a limit of detection of 0.70 parasite equivalents/mL and a limit of quantification of 1.53 parasite equivalents/mL.

Participants’ assessment comprised clinical examination (interrogation and physical examination), electrocardiogram, echocardiogram, and sublingual videomicroscopy.

The analysis of the electrocardiogram was blindly performed by two university cardiologists. The discordant information was analysed by a specialist in electrophysiology.

The patients underwent a 1-dimensional (M-mode), 2-dimensional (mode B) transthoracic echocardiographic study with pulsed, continuous, and colour Doppler (General Electric LOGIQ V2, General Electric Medical Systems, Horten, Norway). All recordings were performed by one investigator blind to the other evaluations, according to the recommendations of the American Society of Echocardiography.^14^

The differentiation between indeterminate CD and Chagas heart disease was based on the presence of electrocardiographic or echocardiographic abnormalities not attributable to other causes.^15^ These alterations included sinus bradycardia with a heart rate < 40 beats/minute, symptomatic sinus node dysfunction, right bundle branch block with left anterior hemiblock, new right branch block, frequent ventricular extrasystoles, abnormal Q waves, second-degree or full atrioventricular block, ventricular tachycardia, left or right ventricular systolic dysfunction (regional or global), ventricular aneurysm , and intracardiac thrombus.

*Video acquisition and analysis* - The patients were studied in the supine position, after 20 min of rest, in a room with a temperature controlled at 22ºC. The microcirculatory network was evaluated in the sublingual mucosa by means of a sidestream-dark-field (SDF) imaging device (Microscan, MicroVision Medical, Amsterdam, Netherlands).^16^ Different precautions were taken and steps followed to obtain images of adequate quality and to ensure satisfactory reproducibility. After careful removal of saliva by isotonic-saline-drenched gauze, the tip of the device was gently positioned in the sublingual area. There was no discomfort associated with the procedure. Steady images of at least 20 sec were obtained while avoiding pressure artifacts using a portable computer and an analog-to-digital video converter (ADVC110, Canopus Co., San Jose, CA, USA). The videos were recorded from three different areas. Video clips were stored as AVI files to allow computerised frame-by-frame image analysis. Files were later renumbered to allow their analysis by a well-trained researcher who was blind to the study allocation. Adequate focus and contrast adjustment were verified, and images of poor quality discarded. The entire sequence was used to describe the semiquantitative characteristics of the microvascular flow and particularly the presence of stopped or intermittent flow. An analysis based on semiquantitative criteria that distinguished between no flow (0), intermittent flow (1), sluggish flow (2), and continuous flow (3) was performed on individual vessels.^17^ The overall score, called the microvascular flow index (MFI), is the average of the individual values.

We used an image-analysis software developed for the SDF-video images (Microscan analysis software^®^-AVA 3.2-MicroVision Medical, Amsterdam, Netherlands)^18^ to determine the total vascular density (TVD). It automatically performs the vessel segmentation (the operation that extracts vessel segments from an image). Then, the user is able to manipulate these intermediate results by deleting, cutting, or connecting vessel segments. If computer-assisted vessel detection fails, one can add remaining vessels by manual tracing with a user-selected diameter. Quantitative red blood cell (RBC) velocity was measured by space-time diagrams.^18^ We also calculated the proportion of perfused vessels (PPV) and the perfused vascular density (PVD) as the total vascular density multiplied by the PPV. The flow heterogeneity index was calculated as the highest MFI minus the lowest MFI divided by the mean MFI.^19^ Heterogeneity was also assessed by means of the coefficient of variation of RBC velocities.

Most of the analysis was restricted to vessels with diameters < 20 μm, whereas the vessels of higher diameter were assessed only for measuring all microvessels TVD and for the detection of compression artifacts. Since flow in large arterioles and venules should be always continuous, the presence of stopped or intermittent flow in those vessels was considered as an evidence of compression artifacts. In addition, compression artifacts were rule out by briefly withdrawing and then advancing the SDF while the pattern of flow was observed. Consequently, videos with such characteristics were not considered for the assessment.

*Data analysis* - Considering the PVD of the sublingual mucosa as the primary measure of outcome and values previously found in healthy volunteers and outpatients with cardiovascular risk factors,^20^ we calculated that 36 subjects were needed in each group to detect a difference of 1 mm/mm^2^ (7%) between volunteers with and without CD, with a power of 80% and a certainty of 95%. Assuming a dropout of 12%, 41 subjects were convoked in each group.

Data distribution was assessed by the D’Agostino-Pearson test. Comparison between groups was performed through unpaired *t*-test or Mann-Whitney, *U*-test for continuous data and Chi-squared test for categorical variables. Data are expressed as mean ± standard deviation (SD), median [0.25-0.75 percentiles] or number (percentage). A p-value < 0.05 was considered statistically significant.

## RESULTS

A total of 166 homes located in Tres Estacas village were evaluated. Nine of them were found with intra-household triatome infestation, 25 with peri-domicile infestation, and two with both. All the 166 homes were treated with beta-cypermethrin.

At the time of the evaluation, 469 inhabitants were found. Of these, 362 agreed to undergo serological studies. Fifty-five of them were positive, resulting in a seroprevalence of 15.2%. Forty-one of the positive cases were > 18 years old. All these patients agreed to participate in the study (n = 41). An equal number of residents with negative serology were convoked. Thirty-eight of them finally participated in the study. All belonged to the Creole ethnicity. The flowchart of the study is shown in Fig. 1.

Table I shows the main epidemiological and clinical characteristics of both groups. The only statistically significant difference was a lower heart rate in patients with CD. None of the patients was symptomatic.

**TABLE I:** Epidemiologic and clinical characteristics of patients with Chagas disease and control group

	Chagas disease (n = 41)	Control group (n = 38)	p-value
Age (years)	42 ± 13	38 ± 14	0.18
Gender, female	22 (54)	25 (66)	0.27
Comorbidities			
Alcoholism	2 (5)	0 (0)	0.17
Smoking	5 (12)	1 (3)	0.11
Arterial hypertension	18 (44)	10 (26)	0.10
Diabetes	5 (12)	3 (8)	0.50
Ischemic heart disease	0 (0)	0 (0)	1.00
Cardiac failure	0 (0)	0 (0)	1.00
Chronic obstructive pulmonary disease	0 (0)	0 (0)	1.00
Arrhythmia	0 (0)	0 (0)	1.00
Syncope	2 (5)	2 (5)	0.96
Venous thromboembolic disease	1 (3)	1 (3)	0.96
Heart rate (beats/min)	69 ± 11	78 ± 16	< 0.001
Systolic blood pressure (mmHg)	130 ± 15	126 ± 23	0.17
Diastolic blood pressure (mmHg)	85 ± 12	83 ± 17	0.39
Mean arterial pressure (mmHg)	100 ± 12	97 ± 19	0.26
Body mass index (kg/m^2^)	31 ± 5	29 ± 8	0.23

Data are shown as mean ± standard deviation or n (%). p-values express the significance of the unpaired *t*-test or *x* ^*2*^ test.

Occurrence of any kind of alterations in the electrocardiogram (one or more) was more common in patients with CD than in the control group (50 vs. 26%, p = 0.03). Differences in specific alterations included longer PR and QT interval duration, and more frequent presence of complete right bundle branch block and complete right bundle branch block with left anterior hemiblock in patients with CD. An abnormal echocardiogram was similarly found in both groups (35 vs. 30%, p = 0.78). Left ventricular posterior wall thickness and right ventricular end-diastolic diameter were more common in CD than in the control group (Table II).

**TABLE II:** Electrocardiographic and echocardiographic findings in patients with Chagas disease and in control group

	Chagas disease (n = 41)	Control group (n = 38)	p-value
PR interval duration (sec)	0.16 ± 0.03	0.15 ± 0.03	0.03
QRS interval duration (sec)	0.09 ± 0.02	0.09 ± 0.02	0.33
QT interval duration (sec)	0.40 ± 0.04	0.37 ± 0.06	0.01
Corrected QT interval duration (sec)	0.42 ± 0.05	0.42 ± 0.03	0.92
Complete right bundle branch block	7 (17)	0 (0)	0.008
Left anterior hemiblock	7 (17)	2 (5)	0.10
Complete left bundle branch block	1 (2)	0 (0)	0.33
Complete right bundle branch block with left anterior hemiblock	4 (10)	0 (0)	0.048
Left atrial diameter (mm)	37 ± 4	36 ± 6	0.47
Left atrial volume (ml/m^2^)	31 ± 8	29 ± 7	0.18
Interventricular septal thickness (mm)	10 ± 2	10 ± 2	0.32
Left ventricular posterior wall thickness (mm)	10 ± 2	9 ± 2	0.03
Left ventricular end-diastolic diameter (mm)	46 ± 6	44 ± 4	0.11
Right ventricular end-diastolic diameter (mm)	32 ± 5	27 ± 4	< 0.0001
Left ventricular systolic dysfunction	2 (5)	0 (0)	0.16
Left ventricular ejection fraction (%)	65 ± 7	66 ± 0.04	0.33
Left ventricular diastolic dysfunction	13 (32)	6 (16)	0.09

Data are shown as mean ± standard deviation or n (%). p-values express the significance of the unpaired *t*-test or *x* ^*2*^ test.

Patients with CD showed increased small microvessels total and perfused vascular density compared with control group. Other microvascular variables did not differ between groups (Table III). Patients with indeterminate disease had a statistically nonsignificant trend to higher microvascular densities than patients with Chagas heart disease [Supplementary data (Table II)]. The same trend was found in the comparison between patients with detected and non-detected RT-PCR [Supplementary data (Table III)]. The microcirculatory variables were similar in patients with CD treated and non-treated with angiotensin-converting enzyme inhibitors or angiotensin receptor blockers [Supplementary data (Table IV)].

**TABLE III:** Sublingual microcirculatory variables in patients with Chagas disease and in control group

	Chagas diseas (n = 41)	Control grou (n = 38)	p-value
All microvessels total vascular density (mm/mm^2^)	22.23 ± 2.19	21.31 ± 2.01	0.06
Small microvessels total vascular density (mm/mm^2^)	20.12 ± 2.33	19.05 ± 2.25	< 0.05
Small microvessels perfused vascular density (mm/mm^2^)	20.03 ± 2.28	19.01 ± 2.25	< 0.05
Proportion of perfused vessels	1.00 [1.00-1.00]	1.00 [1.00-1.00]	0.92
Microvascular flow index	3.00 [2.99-3.00]	3.00 [2.99-3.00]	0.85
Heterogeneity flow index	0.00 [0.00-0.11]	0.00 [0.00-0.11]	0.47
Red blood cell velocity (µm/sec)	973 ± 128	950 ± 130	0.42
Red blood cell velocity coefficient of variation	0.23 ± 0.04	0.24 ± 0.04	0.57

Data are shown as mean ± standard deviation or median [percentiles 0.25-0.75]. p-values express the significance of the unpaired *t*-test or the Mann Whitney *U*-test.

Typical images corresponding to different values of small microvessels total vascular density sublingual microcirculation are shown in Fig. 2 and Supplementary data (video).

## DISCUSSION

To our knowledge, this is the first study assessing *in vivo* sublingual microcirculation performed in patients with CD. Our main result was that patients with CD exhibited increases in total and perfused vascular density of small microvessels compared to non-infected inhabitants of the same village.

In this study, we recruited patients with chronic CD, who mostly had the indeterminate form of the disease. As a group, however, they had lower heart rate and more electrocardiographic and echocardiographic alterations than the control group. They also showed increases in the sublingual total and perfused density of small microvessels. In contrast, we did not find microvascular alterations that could induce ischemia, such as reduced capillary density or increased flow heterogeneity. Consequently, our study suggest that angiogenesis might be the predominant microvascular abnormality in this phase of the disease. These results add a new piece in the growing knowledge about the microcirculatory involvement in CD.

CD, both in its acute and chronic forms, is a systemic illness with widespread multiple organ compromise.^3^ ^,^ ^4^ The complex interactions between the parasite and the immunoinflammatory response might affect the microcirculation.^5^ In mice, acute *T. cruzi* infection is characterised by decreased total and perfused capillary density along with increased leukocyte-endothelium interactions.^7^ These effects seem related to parasitaemia-induced inflammation since benznidazole treatment avoids microvascular brain damage.^21^ In patients with chronic CD, a decreased vascular density was also demonstrated in the myocardium.^22^

Acute and chronic infections may be concurrently associated by the development of angiogenic processes. In short-term models in mice, there are manifestations of exacerbated angiogenesis in the heart, with an increased number of cardiac microvessels.^9^ Local inoculation of *T. cruzi* rapidly leads to angiogenesis in extracardiac tissues.^10^ ^,^ ^23^ Experimentally, angiogenesis involves the expression of proangiogenic factors in the heart; additionally, peritoneal macrophages can increase vascular density in the skin.^24^ In some experimental models, angiogenesis is associated with detrimental effects, such as cardiac remodelling.^9^ A necropsy study performed in patients with Chagas heart disease demonstrated an increased number of perfused capillaries and dilated and tortuous arterioles, together with intense fibrosis.^25^ In contrast, it has been suggested that neovascularisation might be associated with a decrease of cardiac fibrosis. Treatment with a peroxisome proliferator-activated receptor gamma agonist increased angiogenesis and reduced inflammatory mediators and fibrosis in the heart of mice acutely infected.^24^ Calreticulin, a calcium-binding protein present in *T. cruzi* may modulate the complement system and inhibit angiogenesis. This could constitute a potential molecular mechanism for microvascular damage and dysfunction that increases infectivity.^26^ Thus, the increased neovascularisation might delay the pathophysiological mechanisms leading to cardiac symptoms during CD progression.

Angiogenesis is a complex physiological response that, depending on the context, can be either beneficial or detrimental. We could speculate that the indeterminate phase of the disease found in most of the patients in this study, along with the associated increase in microvascular density, might reflect a favourable course of the disease. The non-significant trend to higher microvascular densities in patients with indeterminate disease compared to patients with Chagas heart disease might be in line with this concept.

Similar to our findings in the sublingual microcirculation, an autopsy study identified several vascular alterations in the tongue of patients with chronic CD. These disorders included increases in vascular diameter, vascular wall area, density of the blood vessels, and thickening of the capillary basement membrane.^11^ In another autopsy study, there was correlation in inflammatory damage and reduction in capillary density between cardiac and lingual muscle.^27^ Consequently, the videomicroscopic monitoring of sublingual mucosa might be a tool for the assessment of the microvascular involvement in CD.

We assumed that the increase in microvascular density was an expression augmented angiogenesis. This measurement is commonly used in oncology to assess tumour growth, response to treatment, and prognosis.^28^ ^,^ ^29^ ^,^ ^30^ Thus, in angiogenesis research, accurate, objective, and consistent quantification of microvascular vessels is essential.^31^ For this purpose, we used a validated software-assisted analysis, which is now considered the gold standard for the analysis of images acquired by hand-held videomicroscopy.^18^

Another explanation for the increase in vascular densities might reside in the presence of the capillary recruitment. Capillary recruitment is the active opening of previously closed capillaries in response to higher metabolic demands or tissue hypoxia.^32^ Considering that the patients in this study were clinically stable, the presence of those conditions seems unlikely.

Videomicroscopy allows not only the direct visualisation of microvessels but also an objective quantification of several variables of density (total and perfused vascular density), perfusion (microvascular flow index, proportion of perfused vessels, red blood cell velocity), and heterogeneity (heterogeneity flow index and coefficient of variation of red blood cell velocity). Thus, a detailed analysis of microcirculation may be available from some mucosal vascular beds, including the sublingual area. This information is quite relevant in sepsis and other critical conditions.^12^ In addition, videomicroscopy has been used in many other territories for research and clinical purposes. Beyond sublingual videomicroscopy, there are several approaches to assess tissue perfusion. The assessment of skin perfusion also shows valuable data in some clinical scenarios. Since skin perfusion might not reflect the characteristics of microvascular perfusion in other vascular beds, the combination of both approaches might result in a more thorough characterisation of the microcirculatory disorder.^33^ In patients with CD, however, few studies have evaluated extracardiac tissue perfusion. The use of laser speckle contrast imaging identified an impaired cutaneous microvascular response, but only in patients with reduced left ventricle ejection fraction.^34^ ^,^ ^35^ Since a similar microvascular dysfunction was found in ischemic cardiomyopathy, the disorder might be attributed to the cardiac failure, not to CD.^36^

A strength of our study was the composition of the control group. It included subjects living in the same community, with similar socioeconomic status, ethnicity, diet and environmental exposures as patients with CD. In addition, there were no differences in comorbidities and toxic habits between both groups.

Our study has limitations. The sample size was adequate to demonstrate microcirculatory differences between CD and the control group, but it was insufficient to detect statistically significant differences between patients with indeterminate and Chagas heart disease. The logistics and methodological complexity of the field work was a main difficulty to include a higher number of subjects. Thus, adequately powered studies should address this issue. Although our evaluation was comprehensive in terms of measurements of microcirculatory density, perfusion, and heterogeneity, it lacked other relevant variables, such as the search for adherent and rolling leukocytes in the postcapillary venules.^37^ The characterisation of the inflammatory process at the microvascular level might give further valuable information. In addition, the lack of measurement of angiogenic factors and markers of inflammation is another weakness of the study.

Future directions in research should include the follow-up of our patients with indeterminate and Chagas heart disease, the characterisation of the sublingual microcirculation in the different forms of the disease, as well as the evaluation of microvascular alterations over time and in response to treatment.

*In conclusion* - This prospective observational study showed that patients with chronic CD exhibited increased sublingual microvascular densities. As in other chronic diseases, angiogenesis might be the underlying mechanism. Our findings add further evidence to previous studies carried out in the tongue and in the heart, and suggest that the videomicroscopic assessment of mucosal sublingual microcirculation might be an additional tool for the monitoring of the CD.
