# Supplementary material for: Sublingual microcirculatory alterations in Chagas disease: an observational study in an endemic rural population
Source: Mem Inst Oswaldo Cruz. 2024 Aug 2;119:e240018. doi: 10.1590/0074-02760240018 (PMC11304841; doi:10.1590/0074-02760240018)
Supplement: Supplementary file 1 [file 1678-8060-mioc-119-e240018-s.pdf]

Diagnostic tests

1) Indirect hemagglutination (IHA) (HAI Chagas; Polychaco SAIC, Buenos Aires, Argentina). The specimens were treated with 2-mercaptoethanol and the test was performed according to the manufacturer's instructions. Specimens were considered reactive at a 1:32 or higher dilution.

2) Enzyme-linked immunosorbent assays (ELISA) (The Chagatest ELISA test v.3.0, Wiener Lab, Rosario, Argentina) was used for the qualitative serological screening of anti-*Trypanosoma cruzi* antibodies, following the instructions provided by the manufacturer. In this commercially available assay, antigens are obtained by DNA recombinant techniques starting from specific proteins from the epimastigote and trypomastigote stages of *T. cruzi* strains endemic in South America. Immunoassays based on a native *T. cruzi* strain perform better than those based on non-native strains.[1]

3) Indirect immunofluorescence assay (IFA) was performed using fixed epimastigotes (Laboratorios Chaqueños SA, Resistencia, Chaco, Argentina) and anti-human immunoglobulin G-fluorescein conjugate (Biocientifica, Buenos Aires, Argentina). Specimens were considered reactive when fluorescence was observed at a 1:32 or higher dilution.

4) Multiplex real-time quantitative polymerase chain reaction (qPCR) assay was based on TaqMan technology. The method allows the simultaneous amplification of parasite satellite DNA sequence and a heterologous internal amplification control (IAC) that permits rule out false negative results due to inhibitory substances or loss of DNA during sample processing.[2] Sequences and concentration of primer and probes used are shown in the Supplementary data (Table I).

The amplification was carried out in a Rotor-Gene 6000 (Corbett, UK) or in an Applied Biosystems (ABI 7500, USA) device. The assay has a limit of detection (LOD) of 0.70 parasite equivalents/mL and a limit of quantification (LOQ) OF 1.53 parasite equivalents/mL.

TABLE I

Sequences and concentrations of primers and probes used for the multiplex Taqman real-time quantitative polymerase chain reaction (qPCR) assay

Target	Oligonucleotide	Sequence	Final concentration (µM)
Trypanosoma cruzi satellite DNA	Cruzi 1	59-ASTCGGCTGATCGTTTTCGA-3	0.75
	Cruzi 2	59-AATTCCTCCAAGCAGCGGATA-3	0.75
	Cruzi 3	59-Fam-CACACACTGGACACCAA-NFQ-MGB-39	0.05
IAC	IAC Fw	59-ACCGTCATGGAACAGCACGTA-39	0.1
	IAC Rv	59-CTCCCGCAACAAACCCTATAAAT-39	0.1
	IAC Tq	59-VIC-AGCATCTGTTCTTGAAGGT-NFQ-MGB-39	0.05

TABLE II

Sublingual microcirculatory variables in patients with indeterminate Chagas disease and in patients with Chagas heart disease

	Chagas heart disease (n = 7)	Indeterminate Chagas disease (n = 34)	p-value
All microvessels total vascular density (mm/mm^2)	21.3 ± 1.9	22.4 ± 2.2	0.17
Small microvessels total vascular density (mm/mm^2)	19.0 ± 2.0	20.4 ± 2.3	0.20
Small microvessels perfused vascular density (mm/mm^2)	19.0 ± 2.0	20.2 ± 2.3	0.41
Proportion of perfused vessels	1.00 [1.00-1.00]	1.00 [1.00-1.00]	0.82
Microvascular flow index	3.00 [3.00-3.00]	3.00 [3.00-3.00]	0.92
Heterogeneity flow index	0.00 [0.00-0.00]	0.00 [0.00-0.00]	1.00
Red blood cell velocity (µm/sec)	932 ± 109	981 ± 131	0.20
Red blood cell velocity coefficient of variation	0.22 ± 0.03	0.23 ± 0.04	0.36

Data are shown as mean ± standard deviation or median [percentiles 0.25-0.75]. p-values express the significance of the unpaired t-test or the Mann Whitney U-test.

TABLE III

Sublingual microcirculatory variables in patients with Chagas disease, with detected and non-detected real time polymerase chain reaction (RT-PCR)

	Detected RT-PCR (n = 16)	Non-detected RT-PCR (n = 25)	p-value
All microvessels total vascular density (mm/mm^2)	22.9 ± 2.6	21.8 ± 1.8	0.19
Small microvessels total vascular density (mm/mm^2)	20.8 ± 2.9	19.7 ± 1.8	0.20
Small microvessels perfused vascular density (mm/mm^2)	20.6 ± 2.8	19.6 ± 1.8	0.21
Proportion of perfused vessels	1.00 [1.00-1.00]	1.00 [1.00-1.00]	0.89
Microvascular flow index	3.00 [3.00-3.00]	3.00 [3.00-3.00]	0.82
Heterogeneity flow index	0.00 [0.00-0.03]	0.00 [0.00-0.00]	0.83
Red blood cell velocity (µm/sec)	974 ± 114	972 ± 137	0.96
Red blood cell velocity coefficient of variation	0.23 ± 0.03	0.23 ± 0.04	0.58

Data are shown as mean ± standard deviation or median [percentiles 0.25-0.75]. p-values express the significance of the unpaired t-test or the Mann Whitney U-test.

TABLE IV

Sublingual microcirculatory variables in patients with Chagas disease, treated and non-treated with angiotensin-converting enzyme inhibitors (ACEI) or angiotensin receptor blockers (ARB)

	Treated with ACEI or ARB (n = 16)	Non-treated with ACEI or ARB (n = 25)	p-value
All microvessels total vascular density (mm/mm^2)	22.3 ± 2.4	22.2 ± 2.2	0.86
Small microvessels total vascular density (mm/mm^2)	20.1 ± 2.5	20.1 ± 2.3	0.97
Small microvessels perfused vascular density (mm/mm^2)	19.9 ± 2.4	20.1 ± 2.3	0.82
Proportion of perfused vessels	1.00 [1.00-1.00]	1.00 [1.00-1.00]	0.52
Microvascular flow index	3.00 [2.99-3.00]	3.00 [3.00-3.00]	0.41
Heterogeneity flow index	0.00 [0.00-0.08]	0.00 [3.00-3.00]	0.52
Red blood cell velocity (µm/sec)	988 ± 115	965 ± 135	0.57
Red blood cell velocity coefficient of variation	0.21 ± 0.04	0.24 ± 0.04	0.09

Data are shown as mean ± standard deviation or median [percentiles 0.25-0.75]. p-values express the significance of the unpaired t-test or the Mann Whitney U-test.

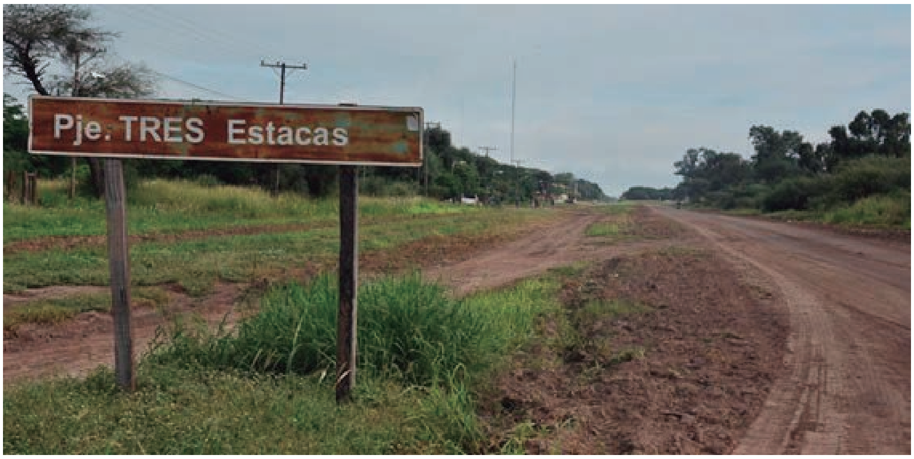

Fig. 1: entrance to the village Tres Estacas.

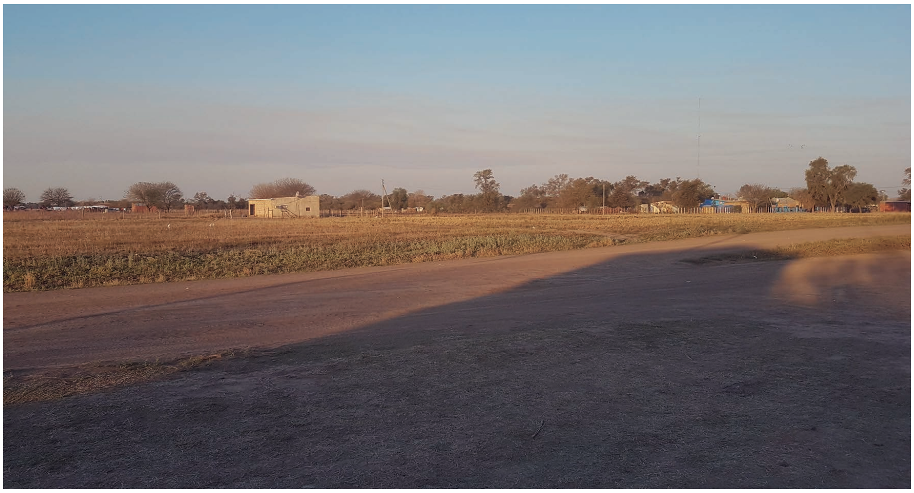

Fig. 2: panoramic view of the village Tres Estacas.

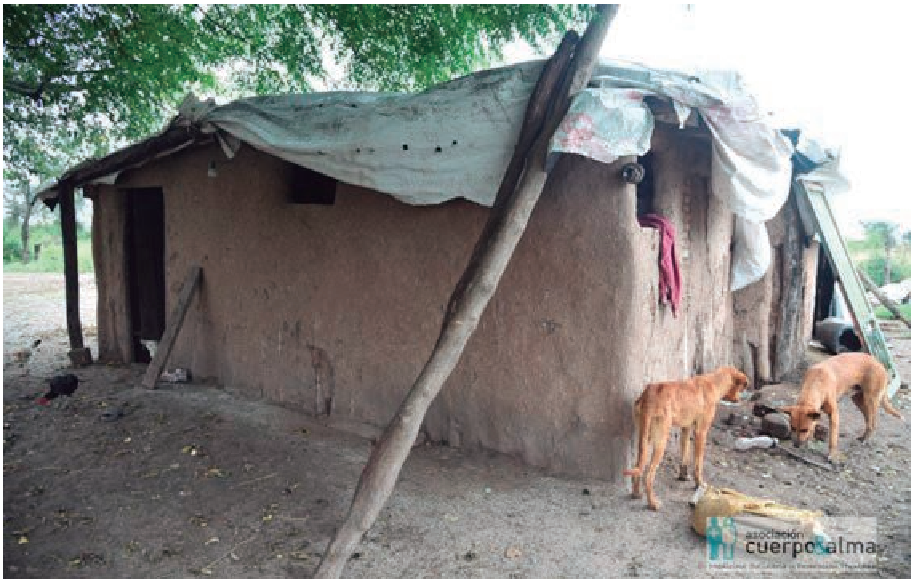

Fig. 3: typical home of the village Tres Estacas.

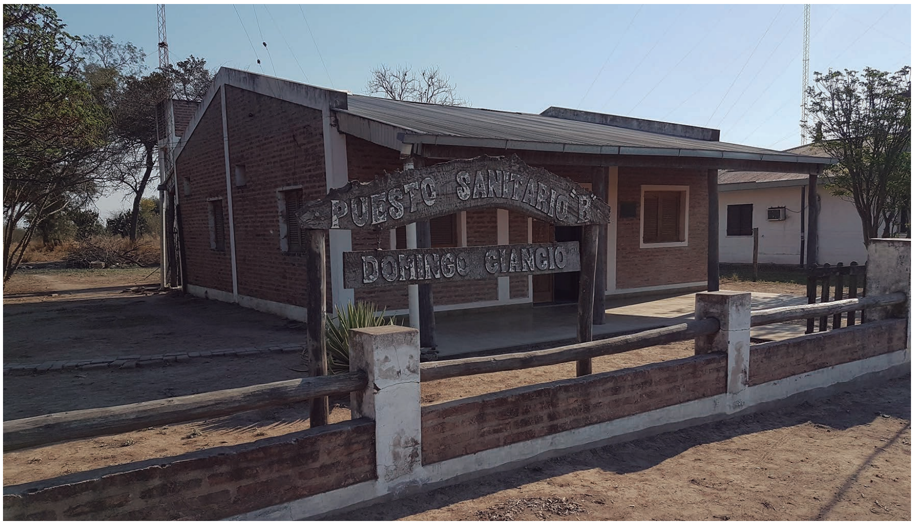

Fig. 4: health post of the village Tres Estacas.

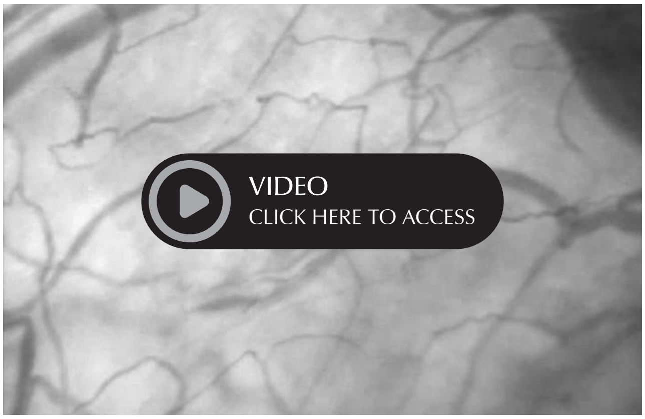

Videos of sublingual microcirculation corresponding to small microvessels total vascular densities of 15.33, 18.93, 20.24, and 22.94 mm/mm2.

REFERÊNCIAS

1. Ballinas-Verdugo MA, Mejía-Domínguez AM, Sánchez-Guerrero SA, Lerma C, Martínez-Cruz M, Álvarez-Manilla-Toquero E, et al. *Trypanosoma cruzi* strain (native or non-native) used as substrate for immunoassays influences the ability of screening asymptomatic blood donors. Rev Invest Clin. 2016; 68(6): 286-91.

2. Duffy T, Cura CI, Ramirez JC, Abate T, Cayo NM, Parrado R, et al. Analytical performance of a multiplex real-time PCR assay using TaqMan probes for quantification of *Trypanosoma cruzi* satellite DNA in blood samples. PLoS Negl Trop Dis. 2013; 7(1): e2000.
